# Dataset for autonomous agriculture using robots to inspect corn and beet

**DOI:** 10.1016/j.dib.2026.112820

**Published:** 2026-05-05

**Authors:** Sergio Sánchez de la Fuente, Luis Prieto-López, Francisco J. Rodriguez-Lera, Ángel Manuel Guerrero-Higueras, Vicente Matellán-Olivera, Miguel Á. González-Santamarta

**Affiliations:** Department of Mechanical, Computer Science and Aerospace Engineering, Universidad de León, Campus Vegazana, s/n, 24007 León, Spain

**Keywords:** Object detection, Crop monitoring, Mobile platforms, Precision farming

## Abstract

Precision agriculture leverages advanced technologies to optimize crop management, increase yield and promote sustainable farming practices. Despite significant progress in agricultural automation, continuous field monitoring remains a challenge for farmers due to labor demands and variable environmental conditions. To address this, the use of mobile robots equipped with intelligent perception systems enables autonomous data collection and analysis in real agricultural environments. This work presents a dataset focused on crop monitoring, containing images of corn and beet fields captured by a ground mobile robot. The images were acquired using the Summit XL platform from Robotnik, equipped with an Intel RealSense D455 camera and collected under natural daylight conditions. The robot was teleoperated across the crop fields while recording rosbags that include RGB images, suitable for tasks such as plant detection. The dataset comprises 10,080 images organized following the YOLO object detection format, with 9104 training images, 493 validation images, and 483 test images. All images are annotated with bounding boxes in normalized YOLO format, distinguishing between two crop classes: beet and corn. To enhance model robustness, the dataset includes augmented versions created through geometric transformations and photometric variations. Privacy protection measures were implemented using automated person detection and anonymization. This dataset aims to support research in precision agriculture, particularly in developing intelligent systems for crop monitoring, plant health assessment, and autonomous agricultural inspection. All data are publicly available through a single Hugging Face repository.

Specifications Table

**Table:** 

Subject	Computer Science
Specific subject area	Object detection dataset for corn and beet crops using an autonomous ground mobile robot to autonomously perform crop inspection and monitoring.
Type of data	2D-RGB Images (.jpg), Ground truth labels (.txt)
Data collection	The images were acquired by using the camera integrated into a ground mobile robot. All images were captured in broad daylight, in a timeframe between 9 am and 1 pm. Specifically, the robot employed in the data acquisition is the Summit XL from Robotnik equipped with an Intel RealSense D455 camera. This robot was teleoperated through crop fields while recording rosbags, which include RGB images.
Data source location	Land in Pobladura de Fontecha 24,250 Pobladura de Fontecha León, Spain Coordinates: 42.421952999999995, −5.6866767 Land in Reliegos de las Matas 24,339 Reliegos de las Matas León, Spain Coordinates: 42.4688501493, −5.3682387952
Data accessibility	• Repository name: Veridis [1] • Data identification number: unileon-robotics/Veridis • Direct URL to data: https://huggingface.co/datasets/unileon-robotics/Veridis
Related research article	None

## 1 Value of the Data

• These data provide real-world RGB images captured by a mobile robot platform in actual agricultural fields, representing authentic environmental conditions and field arrangements. This authenticity makes the dataset particularly valuable for training and validating computer vision algorithms intended for deployment in precision agriculture applications, where laboratory or simulated data often fail to capture the complexity of natural farming environments.
• The dataset enables researchers to develop and benchmark object detection and classification algorithms specifically tailored for corn and beet crops, two economically important agricultural products. The inclusion of ground truth labels allows for supervised learning approaches, facilitating the development of automated crop monitoring systems that can distinguish between different plant species and assess their spatial distribution in the field.
• Researchers in agricultural robotics can utilize this dataset to train perception systems for autonomous navigation and crop inspection tasks. The images captured from the Summit XL robot's perspective provide realistic training data for developing visual servoing, path planning, and obstacle avoidance algorithms that must function in the unstructured environment of crop fields.
• The dataset supports interdisciplinary research at the intersection of computer science, robotics and agricultural engineering. It can be used to develop and validate methods for precision agriculture applications such as yield prediction, contributing to the advancement of sustainable and efficient farming practices.
• The availability of this dataset through an open-access repository promotes reproducibility in agricultural AI research and reduces the barrier to entry for researchers who may not have access to agricultural fields or robotic platforms. This democratization of data accelerates innovation by enabling more research groups to contribute to the development of intelligent agricultural systems.
• Baseline experimental results provided in this work further demonstrate the suitability of the dataset for training and evaluating modern object detection models.

## 2 Background

The increasing global demand for food production, coupled with the need for sustainable agricultural practices, has driven the adoption of precision agriculture technologies. Traditional crop monitoring methods rely heavily on manual field inspections, which are time-consuming, labor-intensive and limited in frequency. This constraint hinders timely decision-making for irrigation, fertilization and pest management. Mobile robotics and computer vision offer promising solutions for autonomous crop monitoring, enabling frequent data collection without human intervention. However, the development of robust perception systems requires datasets captured in real agricultural environments under natural conditions. Previous work has provided foundational datasets for agricultural robotics, such as the sugar beet dataset by Chebrolu et al. [2], while recent advances in deep learning have demonstrated the effectiveness of attention mechanisms for crop classification tasks [3]. This dataset was compiled to address the scarcity of publicly available, robot-perspective imagery of corn and beet crops. The data collection was conducted using the Summit XL mobile robot equipped with an Intel RealSense D455 camera, capturing RGB images during teleoperated traversals through crop fields in León, Spain. The dataset includes annotated images suitable for training object detection models, with the goal of supporting research in autonomous agricultural inspection systems that can operate reliably in unstructured outdoor environments. Recent developments in AI-driven systems for industrial and robotic applications further highlight the importance of data-driven approaches in automation, including hybrid deep learning and intelligent decision-making strategies [7].

## 3 Data Description

The dataset comprises 10,080 images of corn and beet crops captured using the Summit XL mobile robot from Robotnik, equipped with an Intel RealSense D455 camera. The complete data is publicly available at a Hugging Face repository [1]. All images were acquired in agricultural fields located in León, Spain, specifically in Pobladura de Fontecha and Reliegos de las Matas. Detailed acquisition procedures and sensor configuration are described in the Experimental Design section.

### 3.1 Dataset structure overview

The repository is organized following the YOLO object detection format, which is widely adopted in computer vision research and facilitates direct integration with popular deep learning frameworks. The dataset is structured into three main subsets: training, validation, and test sets, with each subset containing paired image and annotation files. This organization enables researchers to immediately begin training and evaluating object detection models without requiring additional preprocessing or format conversion. The directory structure includes separate folders for images and their corresponding label files. This standardized format ensures compatibility with YOLO-based architectures and other modern object detection frameworks that support the YOLO annotation convention.

Total files: 10,080

Structure: Combined dataset in YOLO format:

- train/images/: 9104 training images
- train/labels/: 9104 corresponding train annotation files
- val/images/: 493 validation images
- val/labels/: 493 corresponding validation annotations
- test/images/: 483 test images
- test/labels/: 483 corresponding test annotations

Class mapping:

- Class 0: beet
- Class 1: corn

### 3.2 Individual dataset descriptions

#### 3.2.1 Beet dataset

**Fig. 1:**
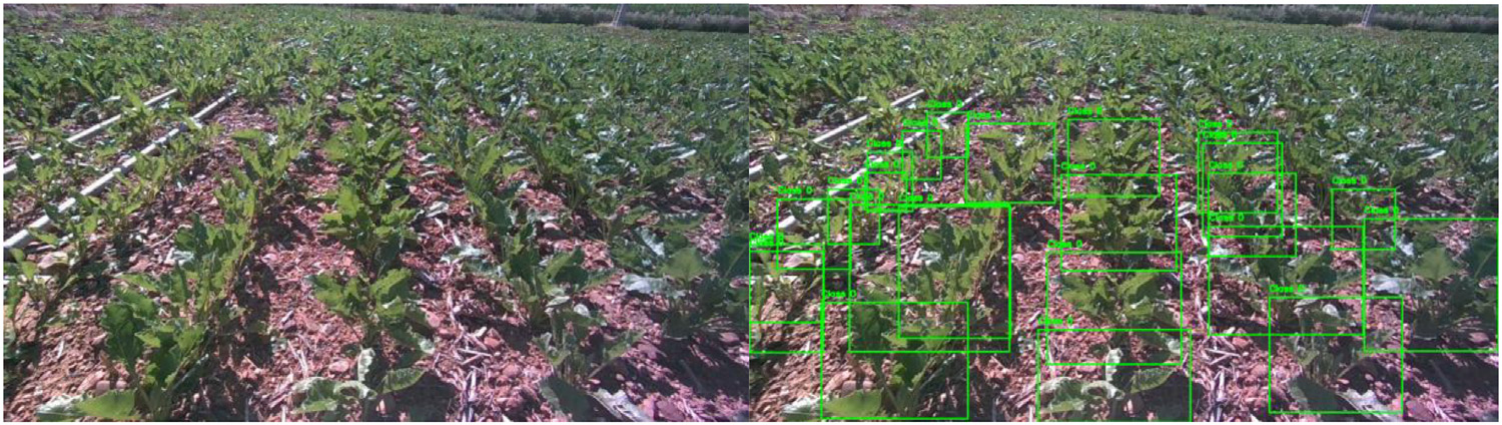
Beet dataset annotation examples. Representative image from the beet dataset showing (left) original RGB image, (center) visualization of YOLO-format annotations, and (right) bounding boxes overlaid on the original image. Images captured by Summit XL robot at ground level with 50 cm row spacing.

The beet directory contains 1605 original images of sugar beet crops captured in agricultural fields. These images were extracted from ROS 2 bag files recorded while the robot traversed beet crop rows. An example of these images is presented in Fig. 1. The beet plants exhibit characteristic broad leaves and the distance between rows of beet is 50 cm. Due to the dense foliage of mature beet plants, some images present significant occlusions where individual plants are partially obscured by neighboring vegetation. The augmented version of this dataset includes 4944 images, generated to increase training diversity and improve model robustness.

#### 3.2.2 Corn dataset

**Fig. 2:**
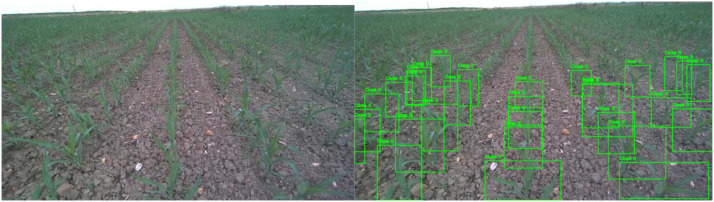
Corn dataset annotation examples. Representative image from the corn dataset showing (left) original RGB image, (center) visualization of YOLO-format annotations, and (right) bounding boxes overlaid on the original image. Images captured by Summit XL robot at ground level with 55 cm row spacing.

The corn directory comprises 1647 original images of corn crops acquired during robot navigation through corn fields. Corn plants present vertical patterns with narrow leaves, making them visually distinguishable from beet crops. An example of the images is presented in Fig. 2, where several crops are shown. In addition, the distance between rows of corn is 55 cm. The augmented version expands this to 5136 images through data augmentation techniques.

### 3.3 Image characteristics and acquisition conditions

All images were captured at a consistent resolution suitable for object detection tasks, maintaining the original aspect ratio from the RealSense D455 camera. Some frames may exhibit varying contrast due to the sun's position relative to the camera angle during robot navigation.

The dataset captures realistic agricultural scenarios with several challenging conditions. For example, natural occlusions occur due to overlapping plants, crop leaves obscuring other plants, and varying crop heights within the same field.

The intrinsic and extrinsic calibration parameters of the Intel RealSense D455 camera are provided in the repository (file realsense_d455_calibration.txt). These parameters allow accurate projection of the 2D YOLO annotations into 3D space using the depth information of the camera.

Although the Intel RealSense D455 sensor provides synchronized RGB-D streams, the publicly released dataset contains only RGB images and YOLO annotations. Depth frames and raw Z-axis information are available in the original ROS 2 bag files, which are also publicly accessible through the Hugging Face repository.

### 3.4 Data augmentation details

The augmented versions of the beet and corn datasets were created to improve model generalization and robustness. The applied augmentation techniques include geometric transformations (rotation, flipping) and photometric variations (brightness, contrast, and saturation adjustments) that simulate different lighting conditions and camera perspectives while preserving the realistic appearance of agricultural scenes.

For each original image, only three random augmentations are applied (not all transformations at once), selected from the following set:

- flip_horizontal: Flips the image horizontally (mirror effect)
- rotate: Rotates the image within a range of −15° to +15°
- adjust_brightness: Adjusts brightness between 0.8× and 1.2×
- adjust_contrast: Adjusts contrast between 0.8× and 1.2×
- adjust_saturation: Adjusts color saturation between 0.8× and 1.2×
- add_noise: Adds random noise to the image
- apply_blur: Applies a light Gaussian blur

These augmentations help models trained on the dataset better generalize to unseen field conditions and camera viewpoints not present in the original data.

### 3.5 Annotation format

All annotations follow the YOLO format (.txt files), where each line represents one object with the following structure:

<class_id> 〈x_center〉 〈y_center〉 〈width〉 〈height〉

Where:

- class_id: Integer representing the object class (0=beet, 1=corn)
- x_center, y_center: Normalized coordinates of the bounding box center (0–1)
- width, height: Normalized dimensions of the bounding box (0–1)

### 3.6 File naming convention

Original images extracted from ROS2 bags follow the naming pattern: rosbag2_<YYYY_MM_DD>-<HH_MM_SS>_frame_<NNNNNN>.jpg

Where:

- YYYY_MM_DD: Date of data collection
- HH_MM_SS: Time of recording
- NNNNNN: Sequential frame number (zero-padded)

### 3.7 Dataset composition

The dataset composition is presented in Table 1. The original set, before augmentation, has 1605 corn images and 1647 beet images. Then, the train set has 1113 corn images and 1163 images, which are increased to 4452 and 4652, respectively, using data augmentation. Besides, the validation set has 251 corn images and 242 beet images, and the test set has 241 corn images and 242 beet images. Thus, the full dataset presents a well-balanced train, validation and test sets.

**Table 1:** Dataset composition, showing the number of images for each class, corn and beet, and for each set, train, validation, and test; taking into account the data augmentation.

Dataset	Total Files	Train Files	Val Files	Test Files
***Beet(Original)***	1605	1113	251	241
***Corn(Original)***	1647	1163	242	242
***Beet(Augmented)***	4944	4452	251	241
***Corn(Augmented)***	5136	4652	242	242
***Mixed***	10,080	9104	493	483

## 4 Experimental Design, Materials and Methods

### 4.1 Data acquisition system

#### 4.1.1 Robotic platform and sensors

The data acquisition was performed using the Summit XL mobile robot platform manufactured by Robotnik Automation. This four-wheeled omnidirectional robot provides a stable base for sensor integration and autonomous navigation in outdoor agricultural environments. The robot was equipped with an Intel RealSense D455 RGB-D camera as the primary sensing device for image capture. The RealSense D455 provides a depth resolution of 1280×720 at up to 90 fps and an RGB resolution of 1280×800 at up to 90 fps. The camera features an 86°×57° field of view for both depth and RGB streams, with an ideal operating range between 0.4 m and 6 m, making it well-suited for ground-level crop monitoring applications. The camera was mounted on the robot at a height of 0.768 m above ground level with a 45° downward inclination angle, positioning it to capture a top-down perspective of the crop rows while the robot traversed the field. This configuration ensured comprehensive visibility of individual plants within the camera's field of view while maintaining sufficient coverage of the surrounding crop structure. During data collection, the robot was teleoperated through the agricultural fields, allowing human operators to guide the platform along crop rows while the sensor system continuously recorded data streams via the ROS 2 framework.

#### 4.1.2 Software framework

The data acquisition system operated on ROS 2 (Robot Operating System 2) Humble distribution. During teleoperation, all sensor data, including RGB images from the RealSense camera, were recorded as ROS 2 bag files (rosbags). The rosbags captured synchronized timestamped data streams, enabling subsequent extraction of individual frames with precise temporal information encoded in the filename structure.

### 4.2 Data collection protocol

#### 4.2.1 Field locations and sessions

Data collection was conducted across 27 field sessions distributed between two agricultural sites in León, Spain:

- Site 1: Pobladura de Fontecha (42.421952999999995, −5.6866767)
- Site 2: Reliegos de las Matas (42.4688501493, −5.3682387952)

Data collection sessions were conducted between 9:00 AM and 1:00 PM on sunny to partially cloudy days, ensuring consistent illumination. The robot was teleoperated across multiple crop rows to achieve broad coverage of variations in crop density, growth stage, and field conditions.

#### 4.2.2 Environmental conditions

The crops exhibited natural variability typical of real agricultural environments:

- Beet fields: Row spacing of 50 cm with dense foliage and significant inter-plant occlusions
- Corn fields: Row spacing of 55 cm with vertical growth patterns and varying plant heights

The 27 data collection sessions were conducted predominantly under stable atmospheric conditions. Weather was generally characterized by full sun, with the exception of a subset of corn acquisition sessions conducted under partially cloudy skies. No significant wind events or precipitation occurred, and all sessions were carried out under dry field conditions.

The crops captured in the dataset correspond predominantly to early growth stages. This is particularly relevant for applications such as early crop monitoring, plant detection, and precision weeding, where plant size and spatial separation are critical factors. Based on the BBCH growth stage scale, the corn plants correspond predominantly to stages BBCH 13–16 (three- to six-leaf unfolded stage), while the beet plants fall within stages BBCH 35–37 (leaf cover of approximately 50–70 %).

### 4.3 Image extraction and preprocessing

#### 4.3.1 Frame extraction from rosbags

Individual frames were systematically extracted from the recorded rosbags using a custom Python script. The extraction was performed at a sampling rate of 1 frame per second to reduce redundancy between consecutive frames. The robot was teleoperated at a low and approximately constant speed during data acquisition, below 0.5 m/s. At a sampling rate of 1 frame per second, this results in a spatial displacement of <0.5 m between consecutive frames. Considering the camera field of view and the crop row spacing (50–55 cm), consecutive frames maintain overlapping visual coverage of the scene. This ensures that relevant plant instances are not missed while reducing redundancy between highly similar frames. Although the Summit XL platform supports a maximum speed of approximately 3 m/s, the teleoperation speed was intentionally kept well below this limit to ensure safe navigation and stable image acquisition within the crop rows.

The extracted images were saved in JPEG format without additional compression beyond the standard JPEG encoding, preserving the original image quality from the camera sensor. Each extracted frame retained its temporal information through a standardized filename convention: rosbag2_YYYY_MM_DD-HH_MM_SS_frame_NNNNNN.jpg, where the timestamp corresponds to the original recording time and the frame number provides sequential ordering within each rosbag.

#### 4.3.2 Quality control and data filtering

Following frame extraction, a manual quality control process was implemented to ensure dataset integrity. Rosbags containing technical errors, such as sensor malfunctions, extreme motion blur, or corrupted data, were identified and excluded from the dataset. This filtering process ensured that only images with acceptable technical quality were included in the final dataset.

### 4.4 Image annotation process

#### 4.4.1 Annotation tool and methodology

All images were annotated using CVAT (Computer Vision Annotation Tool) version 2.25.0 [4], a web-based collaborative annotation platform. The annotation process was conducted by a team of two annotators working collaboratively through CVAT's interface, which enabled efficient annotation workflow and quality assurance.

#### 4.4.2 Annotation guidelines and criteria

The annotation process followed specific guidelines to ensure consistency across the dataset:

Object Selection Criteria: Annotators were instructed to label crop plants that were clearly visible and identifiable as either beet or corn. The primary focus was on plants where the bounding box could encompass the complete or majority of the visible plant structure.

Handling Occlusions: For plants that were partially occluded by neighboring vegetation, annotators prioritized those instances where at least the majority of the plant was visible and clearly identifiable. Heavily occluded plants where identification was ambiguous were initially left unannotated.

Bounding Box Definition: Bounding boxes were drawn to tightly encompass the visible extent of each plant, following the YOLO annotation format with normalized coordinates (center_x, center_y, width, height) relative to the image dimensions.

#### 4.4.3 Annotation workflow and validation

The two annotators worked collaboratively through CVAT's team annotation features, allowing for real-time discussion and consensus on challenging cases. This collaborative approach helped maintain annotation consistency without implementing a formal inter-annotator agreement metric.

After the initial manual annotation phase, a subset of difficult cases, particularly partially occluded plants that were challenging to annotate manually, were processed using a preliminary trained object detection model. This preliminary model was based on the YOLOv11 architecture, trained within the Roboflow environment on a manually annotated representative subset of the dataset, and was used exclusively to generate initial bounding box proposals. To mitigate confirmation bias, all proposals were reviewed by human annotators, who retained full authority to discard or adjust predictions. The model generally failed to detect heavily occluded plants, which were therefore annotated solely by human annotators without model influence.

The completed annotations were exported from CVAT in YOLO format, generating paired .txt label files for each annotated image.

### 4.5 Dataset organization and splitting

The complete dataset was divided into training, validation and test subsets following an approximated 70 %–15 %–15 % split ratio. The splitting was performed at the rosbag level, ensuring that all frames from a given recording session are assigned exclusively to one subset. This rosbag-level splitting strategy prevents data leakage between subsets, as temporally and spatially correlated frames from the same acquisition sequence cannot appear in both training and evaluation sets, while still providing a representative distribution of field conditions and crop appearances across all three subsets.

### 4.6 Privacy protection measures

To ensure privacy compliance and ethical data handling, an automated person anonymization pipeline was implemented to detect and blur any human subjects inadvertently captured in the field images. This pipeline utilized YOLOv11x-seg [5] for person detection and the Segment Anything Model (SAM) [6] with the ViT-H backbone for precise segmentation masks. Detected persons were anonymized by applying Gaussian blur with a kernel size of 51×51 pixels exclusively to the segmented person regions, preserving crop visibility while protecting individual privacy. The anonymization process was applied uniformly across all dataset images before public release. Manual inspection was conducted at the video level on the complete original beet and corn sequences prior to augmentation, which is equivalent to inspecting each extracted frame individually. *Re*-inspection of augmented images was not required, as the applied transformations are purely geometric and photometric and do not introduce new identifiable individuals. No privacy compliance failures were identified during the inspection process.

### 4.7 Code and reproducibility

All custom scripts developed for this dataset are publicly available in the Hugging Face repository alongside the image data. The repository includes a frame extraction script from ROS 2 bags, a data augmentation pipeline with label transformation, and a person anonymization script.

### 4.8 Baseline model performance

To demonstrate the suitability of the dataset for training object detection models, several baseline experiments were conducted using state-of-the-art YOLO architectures, including YOLOv8 (nano), YOLOv9 (tiny), YOLOv10 (nano), YOLOv11 (nano), and YOLOv12 (nano). All models were trained using the predefined training, validation, and test splits provided with the dataset.

The results, presented in Table 2, show consistent performance across different model versions, with mAP@50 values around 0.90 and mAP@50–95 values near 0.60. These results indicate that the dataset provides sufficient variability and annotation quality to support the training of modern object detection models.

**Table 2:** Model performance summary of YOLO models, including YOLOv8 (nano), YOLOv9 (tiny), YOLOv10 (nano), YOLOv11 (nano), and YOLOv12 (nano), trained in the presented dataset. The summary presents precision, recall, mAP@50, and mAP@50–95 for each model.

Model	Precision	Recall	mAP@50	mAP@50–95
**YOLOv8**	0.844	0.874	0.905	0.591
**YOLOv9**	0.844	0.866	0.905	0.587
**YOLOv10**	0.828	0.857	0.897	0.589
**YOLOv11**	0.843	0.871	0.906	0.597
**YOLOv12**	0.850	0.864	0.908	0.595

All experiments were conducted using CUDA 13.0, PyTorch 2.11.0, and the Ultralytics framework (version 8.3.0), ensuring reproducibility of the reported results. Besides, the computer used for the experiments has a RTX 4080 SUPER, 64GB of RAM, and an Intel(R) Core(TM) i9–14900 K.

## Limitations

A limitation of this dataset is the restricted range of illumination conditions. As previously described in the data acquisition protocol, all data were collected between 9:00 AM and 1:00 PM under natural daylight. Consequently, models trained on this dataset may exhibit reduced generalization performance when deployed in environments with significantly different lighting conditions, such as low-light or nighttime scenarios.

## Ethics Statement

The authors confirm that they have read and follow the ethical requirements for publication in Data in Brief. The current work does not involve human subjects, animal experiments or any data collected from social media platforms. Although some images may contain humans, all identifiable features have been blurred to ensure complete anonymization and no human-related data or labels are included in the dataset.

## CRediT Author Statement

**Sergio Sánchez de la Fuente**: Investigation, Data Curation, Writing – Original Draft, Writing – Review & Editing, Visualization, Validation; **Luis Prieto López**: Methodology, Software, Investigation, Data Curation, Visualization, Validation; **Francisco Javier Rodriguez Lera**: Investigation, Resources, Supervision, Funding Acquisition; **Ángel Manuel Guerrero Higueras**: Conceptualization, Supervision, Project Administration; **Vicente Matellán Olivera**: Conceptualization, Supervision, Project Administration, Funding Acquisition; **Miguel Ángel González Santamarta**: Conceptualization, Methodology, Investigation, Data Curation, Writing – Original Draft, Writing – Review & Editing, Supervision, Project Administration, Funding Acquisition.

## Data Availability

Hugging FaceVeridis (Original data)
